# Three-Dimensional Plasmonic Micro Projector for Light Manipulation

**DOI:** 10.1002/adma.201203308

**Published:** 2012-12-04

**Authors:** Chia Min Chang, Ming Lun Tseng, Bo Han Cheng, Cheng Hung Chu, You Zhe Ho, Hsin Wei Huang, Yung-Chiang Lan, Ding-Wei Huang, Ai Qun Liu, Din Ping Tsai

**Affiliations:** Graduate Institute of Photonics and Optoelectronics, National Taiwan University1, Sec. 4, Roosevelt Road, Taipei 10617, Taiwan; Graduate Institute of Applied Physics, National Taiwan University1, Sec. 4, Roosevelt Road, Taipei 10617, Taiwan; Instrument Technology Research Center, National Applied Research LaboratoriesHsinchu 300, Taiwan; Department of Photonics, National Cheng Kung University1, Daxue Road, Tainan 701, Taiwan; School of Electrical and Electronic Engineering, Nanyang Technological University50 Nanyang Avenue, 639798, Singapore; Department of Physics, National Taiwan University1, Sec. 4, Roosevelt Road, Taipei 10617, Taiwan E-mail: dptsai@phys.ntu.edu.tw; dptsai@sinica.edu.tw; Research Center for Applied Sciences, Academia Sinica128, Sec. 2, Academia Road, Nankang, Taipei 115, Taiwan

Applications of surface plasmon polaritons^1^ (SPP) have been extensively developed in the field of optoelectronics, such as plasmonic circuitry (e.g., waveguides, interferometric logic, and modulators),^2^ nanolasers,^3^ ultrahigh-efficiency sensors,^4^ photovoltaics,^5^ super-resolution imaging,^6^ and various two-dimensional plasmonic lens.^7^ Besides, using nanostructures to project SPP plane waves into the adjacent free space is also an important issue. The interactions of plasmonic nanostructure on SPP wave involve not only the in-plane behavior, but also out-of-plane scattering which is captured as the far-field radiated light.^8^ A few theoretical approaches to convert the confined surface plasmons into radiated waves have been proposed.^9^ It is highly desirable to extend the application range of plasmonic devices into the domain of three-dimensional light manipulation.^10^ Recently, three-dimensional focusing and diverging of SPP waves by a quarter circular structure composed of gold (Au) nanobumps were studied.^11^ The forward and backward scattering from individual Au nanobump are observed above and below Au surface, respectively. Hence, the Au nanobumps confer additional three-dimensional propagating wave vectors (*k_x_, k_y_, k_z_*) on SPP wave for departing from surface. Therefore, it is possible to manipulate the three-dimensional plasmonic scattering into specific geometry by arranging the Au nanobumps, which is schematically depicted in **Figure** **1**a. In this paper, we manipulate the scattering of SPP waves by various plasmonic structures composed of arranged nanobumps on a gold thin film. Upon controlling the geometry of the plasmonic structures, the height, position, and pattern of scattered light can be modified as desired. It provides a simple and efficient way to project a specific light pattern into free space, and demonstrate the capability of three-dimensional light manipulation.

**Figure 1:**
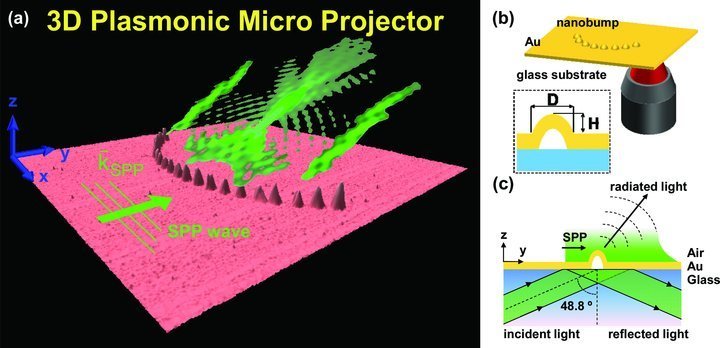
(a) Graphical description of three-dimensional focusing by the propagating beam scattered from an incoming SPP wave impinged on nanobumps structure. (b) Schematic illustration of laser processing for nanobump on the gold thin film. Inset shows the definition of height H and diameter of base D. (c) Graphical description of the scattering from the interactions between SPP wave and a individual nanobump under total internal reflection condition. The individual nanobump is illuminated by the plane wave which is tiled 48.8° from the black-dashed line.

In this experiment, the nanobumps are fabricated on the 30-nm-thick Au thin film on the glass substrate using the femtosecond-laser (fs-laser) direct-writing technique, as depicted in the schematic diagram Figure 1b. Femtosecond-laser direct-writing technique as a kind of maskless fabrication technique is a high-efficiency and powerful method for producing various plasmonic nanostructures.3b, 12 In comparison with other maskless fabrication techniques (e.g., e-beam and focus-ion beam lithographic methods), the processing rate of fs-laser direct-writing technique can be much faster, and the requirements of experimental setup are simple and low-cost. It can be utilized to fabricate structures on arbitrary substrates such as flexible sheets^13^ and optical fibers.^14^ Moreover, with exactly setting the energy dose of the focused laser beam below the ablation threshold of the thin films, the nanobumps can be formed in situ.^15^ The nanobump (the average height H = 30 nm and diameter of base D = 420 nm, see the inset in Figure 1b) is the core in this work. Figure 1c shows the schematic illustration of conversion from SPP wave to radiated light. By total internal reflection microscopy (TIRM), SPP wave is launched by the transverse-magnetic (TM) polarized laser beam (*λ* = 532 nm) under total internal reflection condition (incident angle of 48°). The radiation light is the scattering of SPP wave interacted with a nanobump. **Figure** **2**a shows the TIRM image which is obtained from *z* = 730 nm. The scattering light of SPP wave originates from an individual Au nanobump.^11^ The separation of fringes is 504 nm. To clarify the out-of-plane scattering behavior, the finite difference time domain (FDTD) simulation is used (see simulation method subsection). Figure 2b shows the contour of simulated time-average scattered Poynting field of scattering light from individual nanobump above Au thin film at *z* = 730 nm. This simulation results agree with the experimental optical image. Both the simulation and experimental results show the elliptical intensity contour, which is not the typical dipole radiation (spherical contour in far-filed radiation). The simulated electric-field energy distribution of an individual nanobump under SPP illumination is shown in Figure S1 (Supporting Information). It reveals the electric-field energy distribution at the Au nanobumps is a kind of multipole radiations. The elliptical intensity pattern in experimental and simulation results occurs between the near-field (∼*λ* of incident light) and far-field (>10 *λ* of incident light) zones. Therefore, we conclude from our observations that the radiation pattern cannot simply be described by conventional dipole radiation which is usually launched at the illuminated structure with sizes much smaller than wavelength.^16^ The above results provide the possibility of transformation the SPP wave into the free space by Au nanobump, which is considered as a unique scattering light source for various applications of light manipulation.

**Figure 2:**
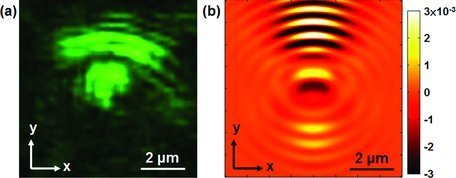
(a) The TIRM image of individual nanobump at the focal plane *z* = 730 nm. (b) The simulated time-average Poynting field contour of scattering light from individual nanobump at *z* = 730 nm.

Next we consider a structure made of a collection of these nanobumps. The various curved arrangements of radii of curvature (RCs) composed of 21 nanobumps with an interspacing of 450 nm are fabricated on the Au thin film, as shown in **Figure** **3**a. The RCs of the structures (*r*) are varied from 2.9 μm to 17.1 μm. The average diameter and height of nanobump are 303 nm and 20 nm, respectively. In TIRM measurement, the focal plane is moved from *z* = -–10 μm (below gold surface) to *z* = +10 μm (above gold surface) for recording the trajectory of scattering light (see Media 1 in Supporting Information, which is the original video clip recorded by a charge-coupled device (CCD) camera in the TIRM system.) Figure 3b is a frame of the acquired original video clip (Media 1) at the focal plane *z* ≍ 0 μm. As shown in Media 1 as well as Figure 3b, the SPP wave propagates along +*y* direction. The curved structures in the left-hand four columns are defined as “concave” relative to the incoming SPP wave, and the reverse curved structures in right-hand side are then obviously regarded as “convex.” Interestingly, the scattered light pattern obtained from structures with various RCs shows clear focusing patterns at different focal planes *z* (see Figure 3c). The focusing patterns are composed of a bright focusing spot with two extended arms. The convex structure shows larger focusing spot size than the concave structure. This difference originates from the different background media: The refractive index (n) of glass substrate and air are 1.5 and 1, respectively. The high-refractive-index material demonstrates higher confinement ability of light in comparison with lower one. Therefore, the spot size focused in the high-index material is smaller than that in the low-index one. This phenomenon has been studied in our previous work.^11^ We therefore define the “scattering-light-focal planes” of the corresponding curved structures at the focal plane *z*, indicating that the brightest focusing spots are observed. For quantifying the relation between the distance from gold surface to scattering-light-focal plane (Δ*z*) and the corresponding RC of the structure, the Δ*z* as a function of the RC *r* is illustrated in Figure 3d. The distance Δ*z* increases with the RC *r* of the structure. Therefore, the RC of the curved structure determines the altitude of the scattering-light-focal planes. In addition, for a closer look at the shape of the light patterns in Figure 3c, both the length of central spot and the separation of two extended arms gradually increase as the RC of curved structure increases. The RCs of the curved structures can control the optical path length, causing the scattered light to form the light pattern at a specific *z* coordinate.

**Figure 3:**
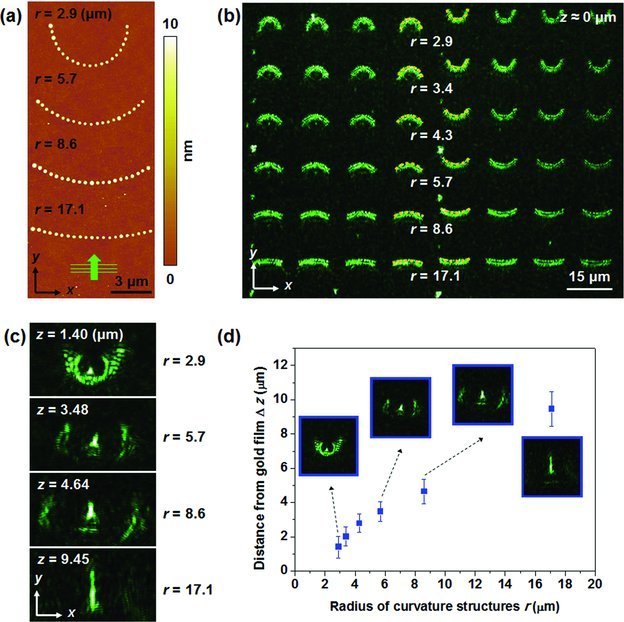
(a) AFM image of the curved structures with radii of curvatures (RCs) r = 2.9, 5.7, 8.6 and 17.1 μm. These structures are all composed of 21 Au nanobumps fabricated on a Au thin film; the interspacing of adjacent nanobumps in each structure is 450 nm. (b) The frame of the acquired original video clip (Media 1) at the focal plane *z* ≍ 0 μm. The four columns at the left-hand (right-hand) side are the scattered light from the concave (convex) structures, which are defined based on the orientation relative to the propagation direction of the SPP wave. (c) TIRM images of the curved structures in (a) recorded at different scattering-light-focal planes *z* where the focusing patterns are observed. (d) The distance (Δ*z*) from the Au thin film to the scattering-light-focal planes as a function of RCs of curved structures. The insets show the corresponding focusing patterns of the curved structures with specific RCs.

As shown in **Figure** **4**a, the five fabricated curved structures (*r* = 5.7 μm and constant arc length) are designed by the adjacent nanobumps with interspacings of 300, 450, 600, 750, and 900 nm on a gold thin film. The TIRM images of convex and concave structures with various interspacings are shown in Figures 4b,c. At the Au surface, i.e., *z* ≍ 0, the bright scattering from each structure is observed. In Figure 4c, at *z* = 3.62 μm, the bright focusing spots with extended arms are observed for the convex structures except for the top case. For the convex structure with 300-nm interspacing, only one focused spot is observed. In addition, the fringe patterns are observed from the concave structures. The intensity profiles of focused patterns from various convex structures are shown in Figure 4d. The intensity of the central peak increases with the reduction of interspacing of adjacent nanobumps. The intensity of central peak of convex structure with interspacing of 300 nm is 6 times as lager as that of 900 nm, as shown in the right-hand-side insets of Figure 4d. The larger intensity of the focusing spot at 300-nm-intersapcing bump structure (in comparison with the structure with larger interspacing) may be associated to the increased number of bumps (considered as scattering centers). The nanobumps can be considered as scattering centers. Therefore, the intensities of the focusing spots are increased with number of bumps (as scattering centers) in single curved structure. The number of arm of each light pattern is also variable with interspacing. Herewith, the interspacing of a curved structure dominates the intensity distribution of the resulting focused pattern. Consequently, the interspacing of adjacent nanobumps is the critical parameter of the *x*-component of scattering wave vector, *k_x_*: the dense interspacing between nanobumps results in stronger multiple scattering.8a The nanobumps at the sides of the curved structure provide the additional propagating wave vectors *k_x_*. We conclude that the Au nanobump structures have uniquely versatile controllability in three aspects: a) Converting SPP wave into far-field light with controllable light patterns. b) Both three-dimensional focusing and defocusing of plasmonic scattering light can be achieved. c) Modulation of plasmonic scattering light can be as far as 20 times in comparison with the wavelength of incident light. Therefore, any specific intensity distribution of the focused pattern can be obtained by properly selecting the interspacing of adjacent nanobumps, indicating the ultrahigh flexibility for our proposed technique.

**Figure 4:**
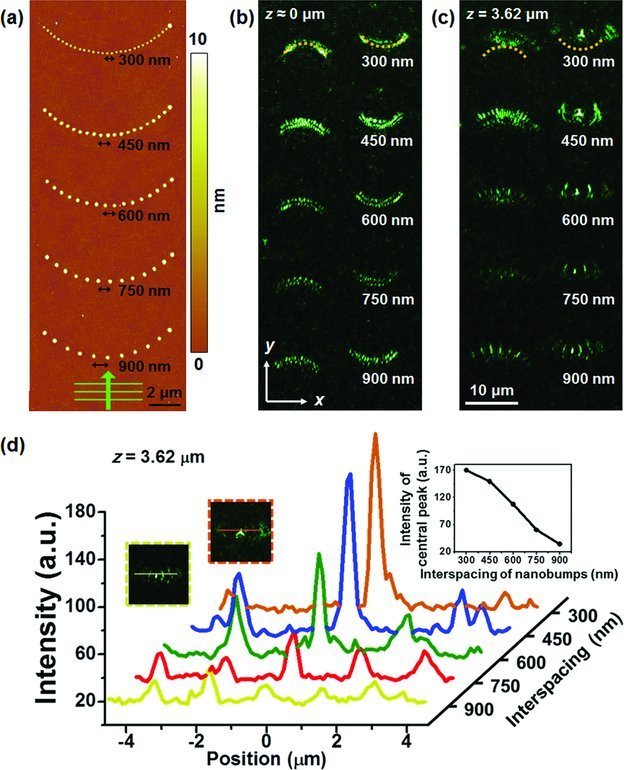
(a) AFM images of curved structures with various interspacings of adjacent nanobumps from 300 to 900 nm. Images (b) and (c) are the TIRM images of the concave (left column) and convex (right column) curved structures with different interspacings at the focal planes of Au film and *z* = 3.62 μm. The SPP wave propagates along the +y direction. (d) Optical intensity profiles of focusing patterns measured from (c) along the cross section lines, which are depicted in upper-left insets. For the convex structure at *z* = 3.62 μm, there are five different curves corresponding to 300- to 900-nm interspacings of adjacent nanobumps, respectively. The upper-right inset shows the intensity of the central peak as a function of interspacings of nanobumps.

By precisely designing a particular curved structure with appropriate RC and adjacent interspacing of nanobumps, we can construct a clear single focusing spot at a specific altitude. As shown in **Figure** **5**a, the structure with RC of 8.6 μm and interspacing of 300 nm is adopted as building block for light projection. Figure 5b shows a single focusing spot in each convex structure at *z* = 4.64 μm, and the FWHM of the intensity profile is 276 nm (inset of Figure 5b). Furthermore, the results show a high consistency of the structures for the shape, intensity, and projection altitude of the focused spot. The curved structures are arranged for projecting a specific optical pattern. Figure 5c shows the TIRM image of the arranged structures at *z* ≍ 0, and the irregular light patterns are observed. However, as shown in Figure 5d, when the focal plane is shifted to *z* = 4.64 μm, the scattering-light-focal plane of the building block, the “NTU” patterns are clearly observed. Moreover, there is a video (Media 2) in Supporting Information that records the light projection trajectory of the scattered light for the designed structures. These results confirm the easy controllability of the focused spot in three-dimensional space by settling the building block.

**Figure 5:**
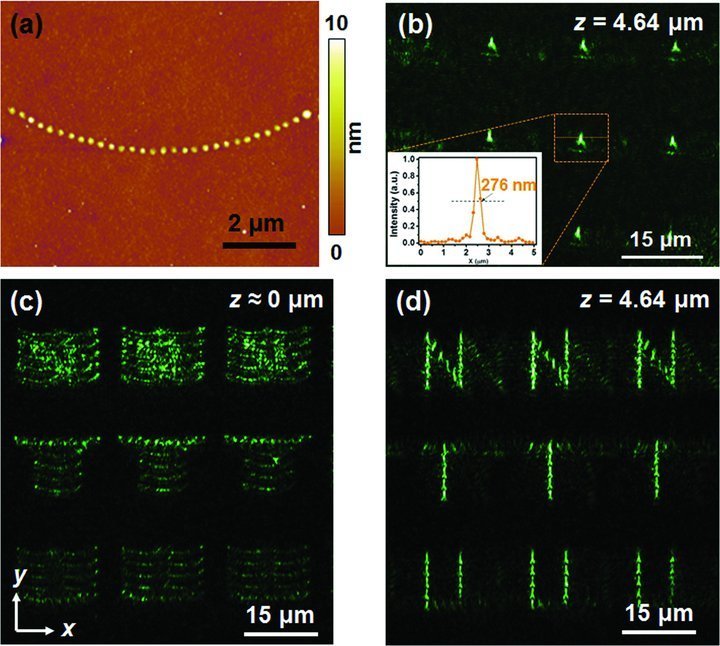
(a) AFM image of the curved structure with an RC r = 8.6 μm and a 300-nm interspacing. Image (b) is the TIRM image of the convex structures at *z* = 4.64 μm. The inset depicted in (b) is the transverse intensity profile of focusing spot of the convex structure. (c) and (d) are the TIRM images of the designed structures, which are arranged by the curved structures in (a), observed at *z* = 0 μm and *z* = 4.64 μm, respectively. The incident SPP wave propagates in the +y direction.

In conclusion, we have reported the out-of-plane plasmonic light manipulation by converting the SPP wave into radiation using various fs-laser fabricated nanobumps on an Au thin film. Both simulation and experiment results of individual nanobump show the unusual scattering radiation, i.e. the elliptical intensity contour. Not only the altitude of the focused light pattern is modulated by adjusting the RCs of curved structures, but also the interspacing of adjacent nanobumps in curved structures determine the intensity distribution of the focused pattern. The RC of curved structure conditions the optical path length and the interspacing of adjacent nanobumps governs the *x*-component of the propagating wave vector. The overall optical results are based on the same principle: interference of light scattered from the SPP waves impinging upon nanobumps. Therefore, the three-dimensional light projection is achieved as long as the arrangements of Au nanobumps are precisely designed. We believe this technique can be further extended to more complicated structures for various applications, including three-dimensional plasmonic circuitry, projection, live-cell imaging, holography, etc.

## Experimental Section

*Simulation*: A TM mode plane wave with wavelength of 532 nm is used to excite the surface wave on Au thin film, and the incident angle is 48.8° (larger than the critical angle), as shown in Figure 1c. The spatial grid size and full computation space are set to 5.0 nm × 5.0 nm × 5.0 nm and 5 μm × 5 μm × 2.5 μm, respectively, and a perfect match layer (PML) is used as the boundaries in the X and Y direction. The relative permittivity of Au is given by the Lorentz-Drude model.^17^ Due to the large size of the simulated region, a distributed memory parallelism and three-dimensional FDTD simulation software MEEP is used.^18^ This simulation environment is simulated by using the Advanced Large-scale Parallel Supercluster (ALPS) computing facilities at the National Center for High-performance Computing in Taiwan.

*Sample preparation*: A 30-nm-thick Au thin film is sputtered on the transparent glass substrate (cover glass, thickness: 0.15 mm). The Au nanostructure are fabricated on Au thin film by a fs-laser fabrication system (central wavelength: 800 nm; pulse duration: 140 fs; repetition rate: 80 MHz). In fabrication process, the laser pulses are tightly focused on the Au thin film by a 100× oil-immersion objective lens with a high numerical aperture (NA: 1.4, working distance: 0.17 mm). The laser power and exposure time are set at 240 mW and 30 ms by an attenuator and a shutter, respectively. Through exact control of the focusing spot, the various arrangements of Au nanobumps could be efficiently fabricated on the Au thin film. The surface morphology of the nonostructure is characterized by an atomic force microscope (AFM, Asylum Research, MFP-3D).

*Measurement*: A total internal reflection microscope (TIRM) system is used to measure the scattering light.^19^ The magnification and NA of oil-immersion objective lens (Olympus, Plan-Apo Oil TIRFM) are 60× and 1.45, respectively. For the excitation of SPP waves, a 532 nm TM-polarized laser beam is illuminated with an incident angle of 48 ± 1°. At this incidence angle, the theoretical surface plasmon wavelength, *λ_spp_*, is 495.2 nm.^20^ For observing the propagation behavior of scattered light, the focal plane of the objective lens for the CCD camera (Hamamatsu Co., ORCA-ER) is adjusted along the *z*-axis to construct the trajectories of the scattered light in the three dimensions.

## Supplementary material

As a service to our authors and readers, this journal provides supporting information supplied by the authors. Such materials are peer reviewed and may be re-organized for online delivery, but are not copy-edited or typeset. Technical support issues arising from supporting information (other than missing files) should be addressed to the authors.
