# Supplementary material for: Three-Dimensional Plasmonic Micro Projector for Light Manipulation
Source: Adv Mater. 2012 Dec 4;25(8):1118–23. doi: 10.1002/adma.201203308 (PMC3613738; doi:10.1002/adma.201203308)
Supplement: Supplementary file 3 [file adma0025-1118-SD3.pdf]

Copyright WILEY-VCH Verlag GmbH & Co. KGaA, 69469 Weinheim, Germany, 2012.

ADVANCED MATERIALS

Supporting Information

for *Adv. Mater.*, DOI: 10.1002/adma.201203308

Three-Dimensional Plasmonic Micro Projector for Light Manipulation

*Chia Min Chang, Ming Lun Tseng, Bo Han Cheng, Cheng Hung Chu, You Zhe Ho, Hsin Wei Huang, Yung-Chiang Lan, Ding-Wei Huang, Ai Qun Liu, and Din Ping Tsai**

ADVANCED MATERIALS

Supporting Information

Three-Dimensional Plasmonic Micro Projector for Light Manipulation

By *Chia Min Chang, Ming Lun Tseng, Bo Han Cheng, Cheng Hung Chu, You Zhe Ho, Hsin Wei Huang, Yung-Chiang Lan, Ding-Wei Huang, Ai Qun Liu,* and *Din Ping Tsai**

[*] E-mail: dptsai@phys.ntu.edu.tw & dptsai@sinica.edu.tw

The electric-field energy distribution of an individual Au nanobump under SPP illumination

For clarifying the origin of the elliptical intensity contour of scattering light from an individual nanobump, the electric-field energy distribution of an individual nanobump is simulated by the three-dimensional FDTD method. A TM polarized plane wave (wavelength: 532nm) tilted with the angle of 48.8° (larger than the angle of total reflection) is used to excite the SPP wave on Au thin film, as shown in Figure 1c. The overall computational region is 800 nm in the *X* and *Y* direction, and 200 nm in the *Z* direction. The grid size along the *X*, *Y*, and *Z* directions all are 1 nm. **Figure S-1** shows the electric-field energy distributions of an individual nanobump in the *XY* plane. For observing the scattered field, both Figure S-1a and S-1b are obtained by point-for-point matrix subtraction of the reference electric-field energy distribution without nanobump from the electric-field energy distribution with nanobump. Figure S-1a and S-1b are the instantaneous electric-field energy distribution at some time *t* and *t+T/4*, respectively, where *T* is the optical period. The time-average electric-field energy distribution, as shown in Figure S-1c, can be obtained by taking averaging of just two instantaneous electric-field energy distribution at some time *t* and *t+T/4*, *i.e.* Figure S-1a and S-1b. Both instantaneous and time-average results show the non-symmetry energy distribution on the individual nanobump, so it implies that the surface charge on the individual nanobump under total internal reflection condition cannot be regarded as the dipole-like symmetric charge distribution.

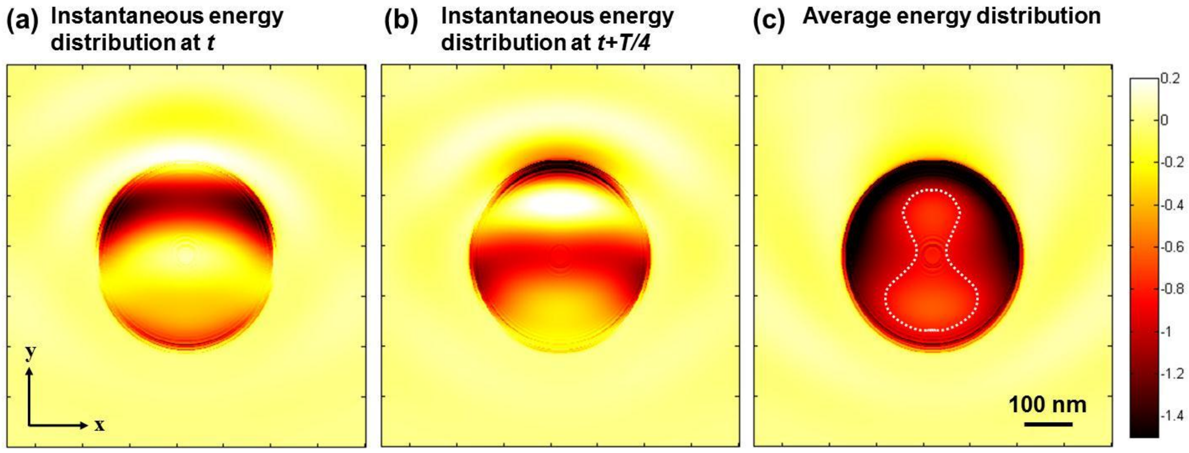

Figure S-1. (a) and (b) are the instantaneous electric-field energy distribution of the individual nanobump on Au film at instantaneous time *T* and *T+T/4*, respectively. (c) is the time-average electric-field energy distribution, obtained by averaging (a) and (b). The SPP wave propagates along *y*-direction.

The trajectories of scattered light from curved structures with different RCs

In our total internal reflection microscope (TIRM) setup for recording the trajectory of scattered light from structures, the focal plane of the objective lens is adjusted from 10 μm below the gold surface, z = –10 μm, to 10 μm above the gold surface, z = +10 μm. In **Media 1**, at first, the focusing patterns of concave structures are observed in sequence for the concaves with RCs from 17.1 to 2.9 μm as the focal plane is elevated. And then, the focal plane approaches $z \approx 0$ μm, *i.e.* Figure 3b, where focusing patterns of concaves and convexes are not observed. After that, the focusing patterns appear one by one for the convexes with RCs from r = 2.9 to 17.1 μm as the focal plane is elevated further above the gold surface. Therefore, the focusing pattern of each curved structure occurs at a specific focal plane. It depends on the radius of curvature of each curved structure.

The trajectory of the light projection from the designed structures

For realizing the three-dimensional light projection, we demonstrate a simple way to present the light projection by a designed structure. The convex structure with the RC 8.6 μm and the interspacing 300 nm is used as a building block to be arranged for three-dimensionally projecting a specific optical pattern in free space. The single focusing spot constructed by the convex structure shown in Figure 5 can be used as a pixel of optical pattern in free space. Therefore, the building blocks are carefully arranged according to the designed pattern by pixels. As shown in **Figure S-2a**, the three-dimensional AFM image shows the structure designed for projecting the letter "N" in free space. The trajectory of the scattered light from the designed structures for three-dimensional projecting in free space is shown in **Media 2**. Figure S-2b to S-3d shows three frames extracted from Media 2 at three specific focal planes. There are concave and convex building blocks marked by yellow-dotted lines. These building blocks are used for the indication of the scattering-light-focal planes where its focusing spots are observed. In Media 2, at first, the fringe scattering light of designed structures composed of building blocks are observed at the focal plane z = –5.34 μm, as shown in Figure S-2b. And then, the focal plane approaches to the Au film, irregular light patterns of the structures are observed, as shown in Figure S-2c. After that, while the focal plane approaches the scattering-light-focal plane z = 4.64 μm, as shown in Figure S-2d, where the focusing spot of the building block is observed, the scattering light is projected into the letters "NTU", as shown in Media 2. These results show the practical way to achieve the imaging projection by nanostructure.

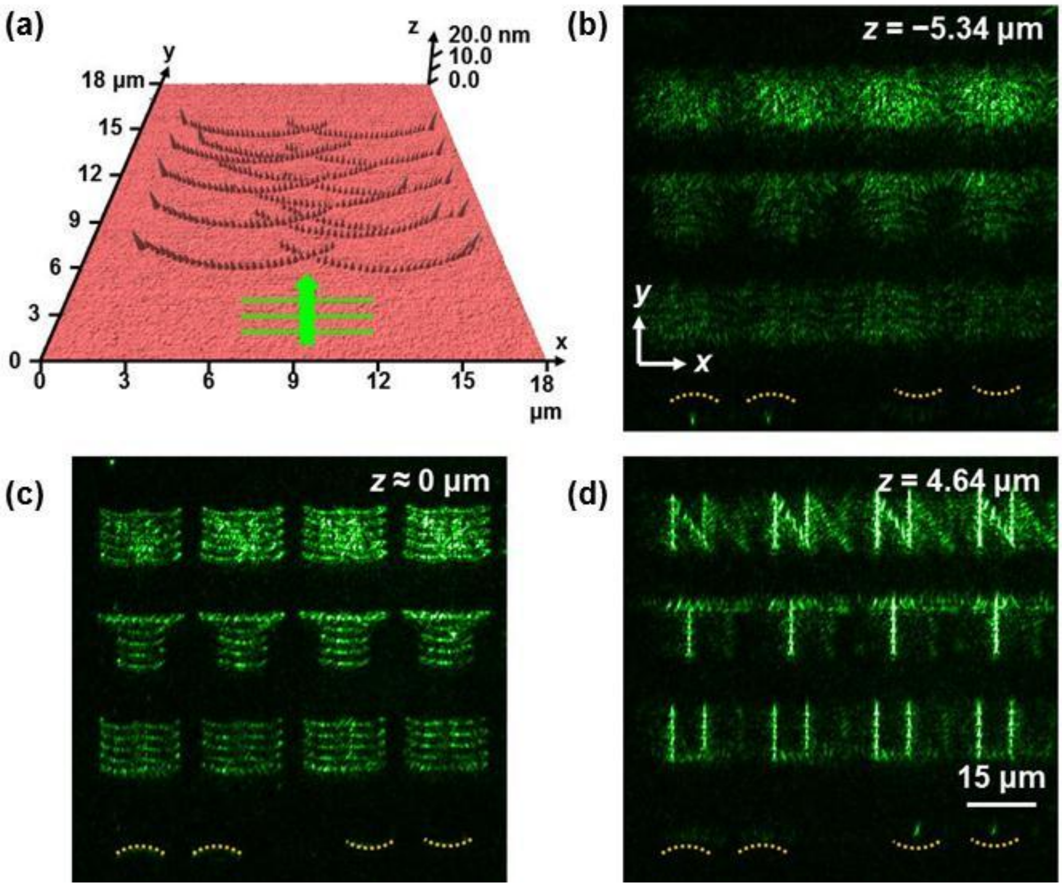

Figure S-2. (a) 3D-AFM image of the designed structure. TIRM images acquired from the original video clip, Media 2 for the trajectory of scattered light from designed structures, at three specific focal planes (b) z = –5.34, (c) z ≈ 0, and (d) z = 4.64 µm.
